# Interrelationship Between Sarcopenia, Frailty and Cardiovascular Disease—The Crucial Role of Obesity in Hypothesis Generation—A Narrative Review

**DOI:** 10.3390/jpm16080422

**Published:** 2026-08-07

**Authors:** Alan Sinclair, Ffion James, Aswani Muraleedharan, Ahmed Abdelhafiz

**Affiliations:** 1 King’s College, London WC2R 2LS, UK; sinclair.5@btinternet.com; 2 Foundation for Diabetes Research in Older People (fDROP), Droitwich Spa WR9 0QH, UK; 3 Department of Geriatric Medicine, Rotherham General Hospital, Rotherham S60 2UD, UK; ffion.james4@nhs.net (F.J.); aswani.muraleedharan1@nhs.net (A.M.)

**Keywords:** sarcopenia, frailty, sarcopenic obesity, cardiovascular, risk, insulin resistance

## Abstract

**Introduction**: Sarcopenia and frailty are emerging independent risk factors for cardiovascular disease. Sarcopenia represents a decline in function associated with reduced muscle mass. Frailty, defined as a phenotype or the multiple stress model, is associated with weakness and a decline in organ reserve with vulnerability to disease. Frailty and sarcopenia may overlap and have shared clinical risk factors including age and malnutrition. **Methods**: We performed a literature review of published studies on frailty and sarcopenia with respect to cardiovascular risk factors and body composition. **Results**: Studies demonstrated that obese sarcopenic or obese frail subjects had a higher prevalence of cardiovascular risk factors such as hypertension, diabetes mellitus, dyslipidaemia, smoking and sedentary lifestyle, which was highly associated with cardiovascular disease. On the other hand, anorexic malnourished frail participants with unintentional weight loss or sarcopenic subjects without obesity had a low prevalence of cardiovascular risk factors, which was less associated with cardiovascular disease. **Conclusions:** Obesity appears to play a crucial role in mediating the cardiovascular risk of both sarcopenic and frail patients.

## 1. Introduction

Cardiovascular disease (CVD) remains the leading cause globally of morbidity and mortality, increasing the burden and costs on the health care systems [1,2]. Besides the traditional cardiovascular (CV) risk factors, there is an increasing interest in sarcopenia and frailty as emerging new risk factors for CVD, especially in older age groups. In addition, sarcopenia and frailty have a combined and progressive effect on CVD [3]. However, most of the studies have addressed sarcopenic or frail participants as single categories without detailed exploration of the effect of body composition. For example, frail older people span across a spectrum from a sarcopenic obese (SO) frail phenotype, with increased insulin resistance, to an anorexic malnourished (AM) frail phenotype with reduced insulin resistance [4]. Therefore, CV risk is likely to be variable across the frailty spectrum due to this variability in insulin resistance. Similarly, sarcopenia combined with obesity, sarcopenic obesity, appears to have an increased CV risk greater than either condition alone [5]. In addition, there is a significant overlap between sarcopenia and frailty, and both conditions commonly coexist [6]. As a result, sarcopenic participants in studies are likely to be frail and vice versa. Therefore, the effect of sarcopenia on CVD risk independent of frailty, or the other way round, is not fully explored or explained. Furthermore, previous studies did not investigate the biological mechanisms of the association between sarcopenia or frailty and CVD. We hypothesise that obesity plays a crucial role in this association by increasing insulin resistance and accelerating the progression of metabolic syndrome. The combination of obesity and the high prevalence of CV risk factors in the sarcopenic or frail patients included in these studies increases the risk of CVD. Therefore, this manuscript reviews sarcopenia and frailty and explores the body composition of the sarcopenic and frail participants to clarify the role of obesity.

## 2. Methods

### 2.1. Data Source

This narrative review searched three databases, PubMed, Google Scholar and Medline, covering recent articles published over the last 10 years from 17th July 2016 to 16th July 2026. We employed Medical Subject Heading (MeSH) terms such as “frail,” “frailty,” “sarcopenia,” “sarcopenic,” “obesity,” “adiposity,” “central obesity,” “central adiposity,” “metabolic syndrome,” “insulin resistance,” “diabetes mellitus,” “body composition,” “cardiovascular disease,” “coronary heart disease,” myocardial infarction “heart failure,” “stroke,” “cardiovascular risk,” cardiovascular events “prediction,” “outcomes,” “adverse events,” and “mortality.” We limited our selection to articles published in the English language. The authors initially screened titles and abstracts for relevance. In addition, the authors conducted a manual search of relevant citations within the retrieved studies to identify additional articles beyond those identified from the electronic search. Within the full text, we searched for reported anthropometric and metabolic characteristics of sarcopenic or frail participants compared with non-sarcopenic and non-frail subjects.

### 2.2. Study Selection

**Figure 1:**
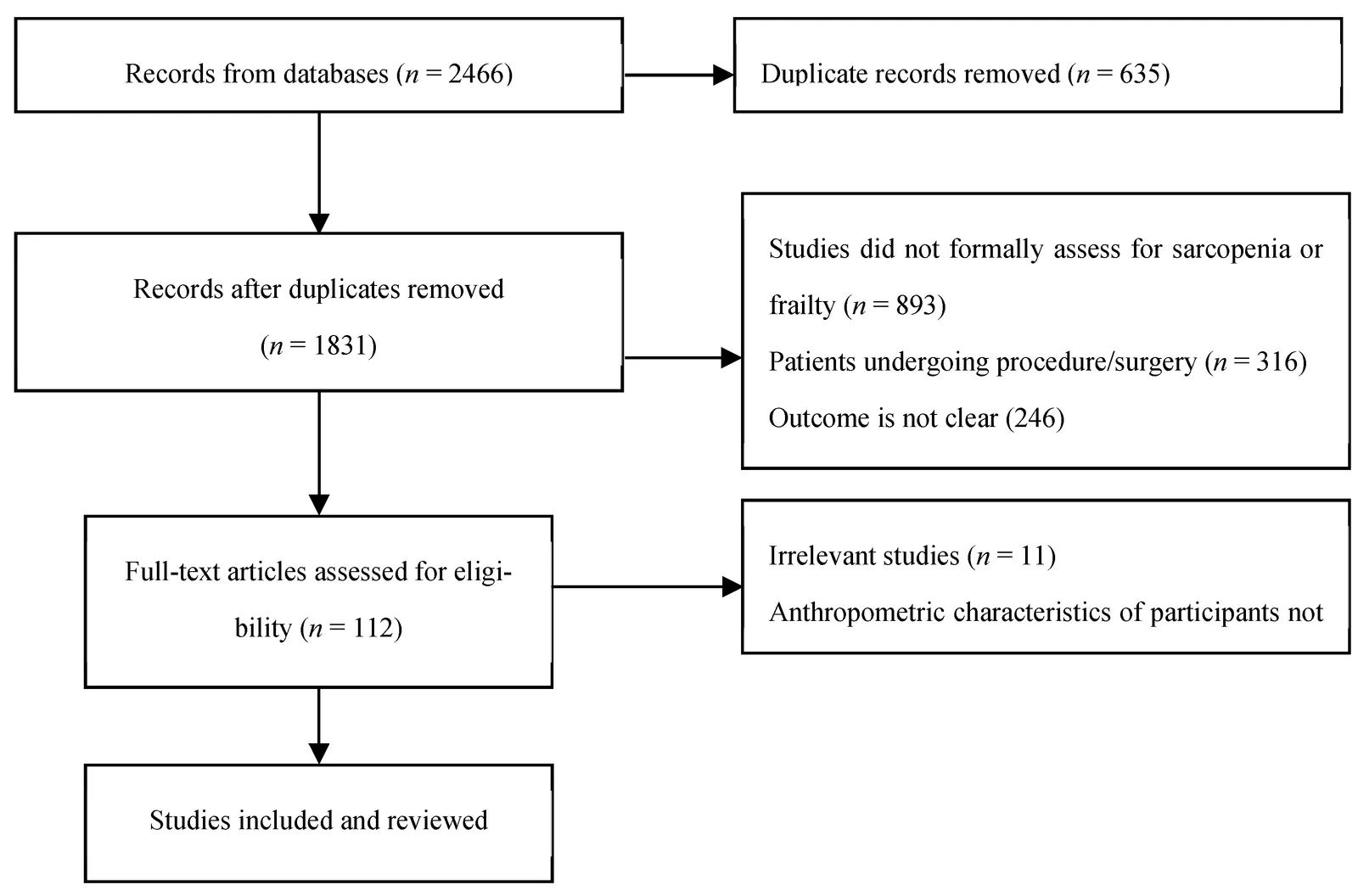
Flow diagram of the studies included.

We searched studies that aimed at investigating the relationship between sarcopenia or frailty and CVD. We included prospective studies that investigated a causal relationship and cross-sectional or retrospective studies that investigated associations. Studies were included if they reported at least all or some of the baseline anthropometric characteristics of sarcopenic or frail participants, such as body weight, body mass index (BMI) and waist circumference. Exclusion criteria included studies that did not report anthropometric data, non-English language articles, studies conducted on patients without a clear sarcopenia or frailty diagnosis, reviews, editorials, abstracts, conference proceedings, non-human studies or expert opinions. A flow diagram of the included studies is illustrated in Figure 1.

### 2.3. Data Extraction

The authors independently reviewed the studies and extracted data into a standardised table format. For each study, data were extracted into the following 5 categories:

1. Study, which includes author, study design, country of origin and year of publication.
2. Population, which included number, mean age and duration of follow-up.
3. Aim of the study.
4. Body composition, including baseline anthropometric and metabolic characteristics of sarcopenic or frail subjects compared with non-sarcopenic or non-frail subjects. These included data such as body weight, BMI, waist circumference, lipid profile, diabetes mellitus, hypertension or other metabolic characteristics.
5. Outcomes, which included CV outcomes by sarcopenia or frailty status.

The authors are aware that this search strategy could be limited by missing contributions from non-English published articles. We resolved any disagreements by detailed discussion and review of any conflicts in data interpretation to reach mutual agreement.

## 3. Sarcopenia

Sarcopenia is defined as a progressive loss of muscle mass, strength and function, which may lead to an increased risk of falls, disability and mortality [7]. The European Working Group on Sarcopenia in Older People 2 (EWGSOP2) and the Asian Working Group for Sarcopenia (AWGS) are the most commonly used tools for sarcopenia diagnosis [8,9]. The EWGSOP2 defined probable sarcopenia when low muscle strength is detected, confirmed sarcopenia when low muscle quantity or quality is present and severe sarcopenia when low muscle strength, low muscle quantity/quality and low physical performance are all detected [8].

The Asian working group have simplified the diagnostic elements to low muscle mass combined with low muscle strength and physical performance, which is considered an outcome indicator [9]. The risk of sarcopenia is related to older age, decline in sex and growth hormones, low body weight and poor nutrition [10]. Due to muscle mass loss, inadequate nutrition and reduced protein intake, sarcopenia is associated with other related nutritional conditions such as malnutrition and cachexia. The three conditions share interrelated pathophysiology that leads to different magnitudes of wasting [11]. However, with the increasing prevalence of obesity, sarcopenia can be associated with obesity, a condition called sarcopenic obesity (SO) [8]. Sarcopenic obesity is defined as increased visceral fat that concurrently exists with decreased muscle mass, strength and function [12]. There is still a lack of standardised diagnostic criteria for SO, which may influence its clear clinical identification, epidemiological comparison and clinical implications. This is likely due to the difficulties associated with a unified diagnostic criteria of sarcopenia [13]. Although there are wide variations in the diagnostic criteria of SO, it can be defined as subjects fulfilling both the criteria for obesity and the EWGSOP2 or Asian working group criteria for sarcopenia. Previous studies confirmed the association between SO and prevalent CV risk factors such as insulin resistance, adverse glucose metabolism, dyslipidaemia, hypertension and metabolic syndrome compared with sarcopenia or obesity alone [14].

## 4. Frailty

Frailty is defined as an increased vulnerability to psychological and physical stressors because of reduced physiologic reserve at multiple organ levels [15]. It is a progressive condition, which leads to falls, injuries, disability, institutionalisation and mortality [16]. Therefore, frailty is likely to be a pre-disability state although it can coexist with disability [17]. Although there are many tools for frailty assessment, they are largely based on either the phenotype or the cumulative deficits approaches. The most well-validated and widely used are Fried’s criteria for the phenotype and Rockwood’s frailty index (FI) for the cumulative deficits [18,19]. Fried’s criteria include unintentional weight loss, muscle weakness, slowness, low physical activity and exhaustion. Absence of these criteria indicates robust state, presence of 1–2 indicates pre-frailty and presence of ≥3 indicates frailty. The Rockwood’s frailty cumulative deficits approach is based on age-related medical, functional and psychosocial deficits that constitute the FI. The FI counts up to 70 clinical deficits mapped into the interval between 0 and 1. The greater the number of deficits, the higher the FI and likelihood of adverse outcomes. The phenotype approach views frailty as a syndrome, which better predicts disability, while FI views frailty as a spectrum, which better predicts mortality. The frailty phenotype is more feasible for screening, whereas FI is better suited for management and follow-up. So far, there is no gold standard frailty assessment tool, and the existing ones are variable in their capabilities of clinical assessment and prognostic benefits [17].

## 5. Sarcopenia–Frailty Overlap

**Figure 2:**
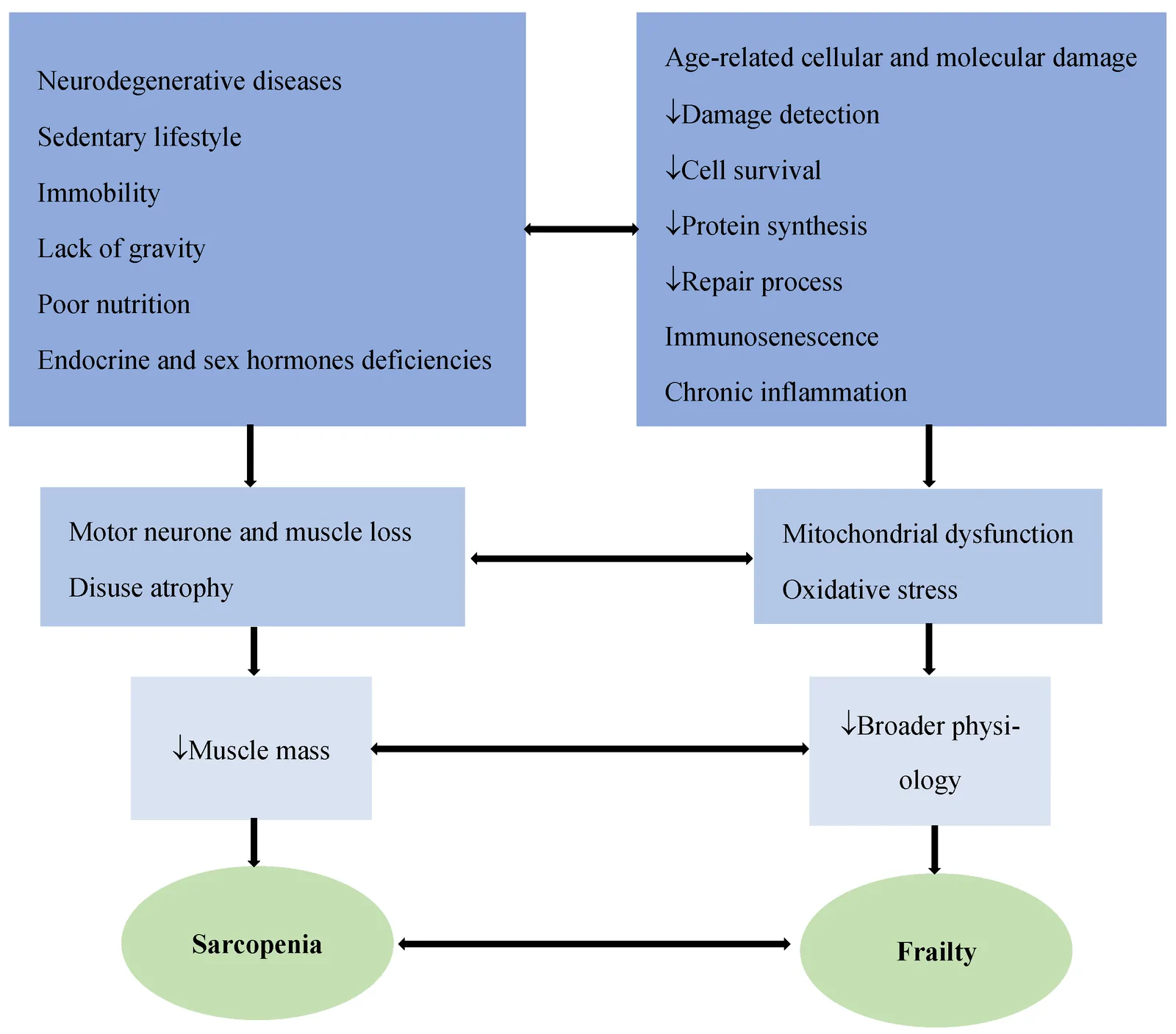
The significant overlap in the pathogenesis of sarcopenia and frailty.

There is a significant overlap between sarcopenia and frailty, especially when reduced muscle strength, low gait speed, slowness and exhaustion are considered. If frailty is defined as a syndrome at multiple organ levels, sarcopenia is defined as a disease at one organ level. In other words, sarcopenia can be viewed as the muscular manifestation of frailty, or, put simply, as ‘muscle frailty’. Sarcopenia is often an intermediate step in the development of frailty, and both conditions share common aetiology, pathogenesis, symptoms and clinical outcomes [20]. Therefore, most frail patients will have sarcopenia and vice versa [6]. Chronic inflammation, mitochondrial dysfunction and oxidative stress are shared pathogenic mechanisms (Figure 2) [21]. Because weight loss is not a prerequisite for frailty diagnosis, frailty can be associated with obesity. Therefore, frailty also overlaps with SO. We hypothesise that sarcopenia–frailty syndrome is a continuum or a spectrum that has an SO frail phenotype at one end and an AM frail phenotype at the other end. The SO frail phenotype is associated with an unfavourable metabolic profile, with increased insulin resistance and increased prevalence of CV risk factors. On the other hand, the AM frail phenotype is characterised by malnutrition and significant weight loss, which leads to reduced insulin resistance and reduced prevalence of CV risk factors. The synergistic effects between reduced muscle mass and increased adipose tissue mass lead to cardiometabolic disease and increased risk of CV events mostly in the SO frail phenotype. In clinical studies, both sarcopenia and frailty have been shown to increase the risk of CVD. We hypothesise that the participants included in these studies were either overweight or obese, with an unfavourable metabolic profile that increases the risk of CVD. We also hypothesise that sarcopenic or frail people with significant weight loss (shrinking syndrome) or the AM frail phenotype are unlikely to have persistent increased risk of CVD, due to the regression of metabolic syndrome and reduced prevalence of CV risk factors in this vulnerable population.

## 6. Studies with High BMI Participants

The sarcopenia and frailty studies, which included high BMI participants, are summarised in Table 1. Most of the studies included sarcopenic participants with higher body weight than non-sarcopenic subjects. In addition, the prevalence of metabolic syndrome and CV risk factors were more prevalent in sarcopenic than non-sarcopenic participants. Yu et al., in their prospective study, reported that sarcopenia increased the risk of CVD, CV mortality and all-cause mortality. However, the sarcopenic participants in this study had higher BMIs and more prevalent hypertension, diabetes mellitus, dyslipidaemia, chronic kidney disease (CKD) and underlying CVD than non-sarcopenic participants [22]. Boonpor et al. reported similar results of sarcopenia increasing the risk of CVD only in obese subjects [23]. Xin et al., in their large prospective study, showed that sarcopenic overweight subjects have an unfavourable metabolic profile and increased prevalence of CV risk factors compared to normal weight sarcopenic participants. The CV risk increased only in sarcopenia with general obesity, sarcopenia with abdominal obesity and sarcopenia with being overweight [24]. The definition of obesity may have variable results in different studies. For example, Jiang et al. demonstrated in a prospective study that SO increased the risk of CVD when obesity was defined based on BMI or waist circumference [25]. Chuan et al. showed that SO, with obesity defined based on percentage of body fat, increased the risk of CVD compared to sarcopenia without obesity or obesity without sarcopenia. Obesity, based on BMI, was not useful in calculating SO [26]. In Fukuda et al.’s study, SO increased the risk of CVD when obesity was defined based on android to gynoid ratio or android fat mass. Obesity defined using total body fat or BMI did not show an association with CVD [27]. Farmer et al., using data from a large biobank study, demonstrated that obesity was associated with CVD and muscle quality improvement did not improve outcomes [28]. Similar to sarcopenia studies, frailty studies included frail participants with higher body weight than non-frail subjects. In addition, the prevalence of metabolic syndrome and CV risk factors was higher in frail or pre-frail subjects compared with robust subjects. Hannan et al., in their prospective study, found that frailty assessed by Fried’s criteria increased the risk of CV events. Frail and pre-frail subjects were more obese and had a higher prevalence of CV risk factors compared with robust subjects [29]. He et al. investigated the changes in the frailty status and its effect on CV risk in three subject cohorts. The progression to frailty increased, while regression of frailty decreased the risk of CVD. Frail participants in all cohorts had higher BMI and unfavourable metabolic profiles compared with robust participants [30]. Chen et al. showed that pre-frailty and frailty, assessed by Fried’s criteria, prospectively increased the risk of CVD by 15%. Pre-frail and frail subjects had higher BMIs and used more medications for diabetes, hypertension and lipid-lowering compared with robust participants [31]. Similarly, Damluji et al. reported that frailty, assessed by Fried’s criteria, increased the risk of major adverse cardiovascular outcomes (MACE) in obese pre-frail and frail participants with prevalent CV risk factors [32]. Veronese et al., using Fried’s criteria, demonstrated that frailty increased the risk of incident CVD in obese frail subjects with metabolic syndrome [33]. Fan et al. found that every 0.1 increment in FI increased the risk of CV mortality. In comparison with robust subjects, pre-frail and frail participants had a higher waist-to-hip ratio, indicating central obesity [34].

## 7. Studies with Low BMI Participants

The sarcopenia and frailty studies that included low BMI participants are summarised in Table 2. Studies reported that sarcopenia alone, without associated obesity, did not increase the risk of CVD. For example, Chen et al. demonstrated that both sarcopenia and severe sarcopenia were not associated with CVD. The sarcopenic and severe sarcopenic participants had lower BMI, body weight and no obesity compared with non-sarcopenic subjects. On the other hand, likely sarcopenic subjects with higher BMI, body weight and central obesity had an increased risk of new-onset CVD [35]. Similarly, Zeng et al. showed that sarcopenia in subjects with low BMI did not increase the risk of CVD [36]. Gao et al. reported that low muscle mass alone or being underweight did not increase the risk of CVD [37].

Studies that included non-obese frail participants did not show an association of frailty with CVD. For example, Patel et al. demonstrated that although there was an association of frailty with all-cause mortality, frail subjects had a similar weight to non-frail subjects and frailty was not associated with CV mortality [38]. Similarly, Kleipool et al. reported no association between frailty, assessed by Fried’s criteria, and incidence of CVD. Frail subjects had lower body weight, total cholesterol and low-density lipoprotein (LDL) levels compared with non-frail participants [39]. Zhu et al. explored the association of the circadian syndrome (CircS) combined with frailty, assessed by Fried’s criteria, and CVD. Participants with both CircS and frailty were more likely to have new-onset CVD compared with either condition alone. Frailty on its own was not significantly associated with incidence of CVD. Frail subjects had a significantly lower BMI compared with CircS alone or the combined CircS and frail participants [40].

## 8. Baseline CV Risk

In both sarcopenia and frailty, obesity appears to be a crucial factor in determining CV risk. Obesity is associated with increased adipose tissue deposition in internal organs, including skeletal muscles. Therefore, an increase in muscle mass will be required to counterbalance the excess intramuscular deposition of fat to maintain muscle mass–adipose tissue mass balance [12]. The reduction in the muscle mass–adipose tissue mass ratio leads to an imbalance in cytokine homeostasis, with a decrease in the anti-inflammatory muscle-secreted myokines and an increase in the pro-inflammatory adipose tissue-secreted adipokines. This leads to a state of chronic low-grade inflammation, oxidative stress and mitochondrial dysfunction. Because skeletal muscle is the largest insulin-dependent glucose disposal organ, the accumulation of intramuscular adipose tissue leads to increased insulin resistance [41]. This inflammatory state, combined with increased insulin resistance, leads to endothelial injury, atherosclerosis and, therefore, increased CV risk [42,43]. Furthermore, insulin resistance causes hyperglycaemia, which leads to dyslipidaemia and fatty liver. This elevates the levels of free fatty acids, which further increase visceral fat deposition, setting a vicious circle of unfavourable metabolism, which augments the risk of mortality [44,45]. In addition to the above, lifestyle factors such as physical inactivity and sedentary lifestyle promote a muscle-depleting obesity, which contributes to the aetiology linking SO to CVD [22]. Obesity is an important factor in mediating the relationship between sarcopenia and CVD, as obesity per se is a CV risk factor, and this effect is synergistically enhanced by the presence of sarcopenia [22]. A recent Japanese study has shown that a combination of low handgrip strength and obesity significantly increased the risk of CVD {hazard ratio (HR) 1.49, 95% confidence interval (CI) 1.03 to 2.17} [46]. There may be an interaction or a cumulative effect of obesity and sarcopenia. It has been shown that subjects with both sarcopenia and obesity had a 24% (95% CI 12% to 7%) higher risk of all-cause mortality compared with individuals with either condition alone [47]. Similar to sarcopenia, frailty studies, which showed an association of frailty with CVD, included participants who were either overweight or obese with a high prevalence of metabolic syndrome and baseline CV risk compared with robust individuals [29,30,31,32,33,34].

The presence of sarcopenia without associated obesity does not appear to increase the risk of CVD [35,36,37]. For example, in one study, the proportion of overweight subjects in the definite or severe sarcopenia group was <5.0% and this group was not associated with new-onset CVD. In contrast, the proportion of overweight subjects in the possible sarcopenia group was >50.0%, and this group was associated with CVD [35]. This may also suggest that the progression from possible sarcopenia, being overweight, to definite or severe sarcopenia, with weight loss, may attenuate the vascular risk-potentiating effects of obesity and mitigate the increased risk of CVD [36]. Other studies showed that low muscle mass alone was not correlated with increased risk of CVD, and authors suggested that this might be due to the confounding factor of body fat mass [28,37,48]. In one study, there was no significant statistical association between sarcopenia and incident CVD in participants with cardio-kidney-metabolic (CKM) disease stage 0, likely due to the absence of metabolic or CV risk factors in these participants. In contrast, the association between sarcopenia and incident CVD was amplified among participants with metabolic and CV risk factors at CKM stages 1–3 [49]. In another study, higher CKM disease stages were associated with greater probabilities of progression to more severe sarcopenia states in subjects who were more obese and had more prevalent CV risk factors than those without sarcopenia. This shows the significant association of sarcopenia with unfavourable metabolism that increases the risk of CVD [50].

Similar to sarcopenia, frailty alone without obesity or metabolic syndrome appears not to increase the risk of CVD. For example, one study including frail subjects with lower body weight, low diastolic blood pressure and low cholesterol levels showed that frailty was not associated with CVD. This cohort of participants is likely to have a high level of malnutrition, regression of metabolic syndrome and a low prevalence of CV risk factors, and therefore a lower risk of CVD [39]. Another study has shown that low BMI frail subjects have no increased risk of incident CVD. The study demonstrated that frailty increased the risk of new-onset CVD only when metabolic syndrome and obesity were present. This suggests that increased CV risk factors such as dyslipidaemia, hypertension and hyperglycaemia are likely to play a pivotal role in the risk of CVD, whereas frailty associated with a healthy metabolic profile may not predict CV risk [40]. In one study, there was a positive association between FI and CVD, being more evident in subjects with BMI ≥25 without prevalent CV risk factors such as hypertension, diabetes mellitus or dyslipidaemia, confirming the important role of obesity [51]. In addition, although one study showed an association of frailty components with CVD, unintentional weight loss was not significantly associated with the risk of CVD (HR 1.45, 95% CI 0.92 to 2.29, *p* = 0.11) [33]. Unintentional weight loss did not predict CVD in another previous study [52]. Furthermore, shrinking syndrome (defined as low BMI < 20) was not associated with incidence of CVD, fatal CVD, MACE, myocardial infarction, hospitalisation for heart failure or stroke in the post-hoc analysis of the ASPREE trial [53].

## 9. Clinical Implications

We conclude that CVD risk associated with sarcopenia or frailty is likely related to the associated obesity and the prevalent metabolic syndrome and CV risk factors such as hypertension, diabetes mellitus and dyslipidaemia. The increase in general and central obesity combined with the decrease in muscle mass and muscle strength leads to a state of metabolic dysregulation of fat–muscle balance, which is associated with CVD. Therefore, treatment should focus on promoting regular physical activity and balanced nutrition to assist with maintenance of optimal body composition from adulthood to older age. A study found that achieving ideal cardiovascular health (CVH) metrics, defined as three core health behaviours (smoking cessation, physical activity and balanced diet) and four health factors (ideal body weight, low cholesterol, normal blood pressure and controlled glycaemia), was significantly associated with a lower risk of all CVD outcomes in frail subjects. On the contrary, frailty associated with poor CVH led to a higher risk of CV outcomes [31]. Not only is prevention of sarcopenia and frailty required, but also the reversal of the possible sarcopenia and pre-frailty to the robust state may reduce the risk of developing CVD [30]. The definition and diagnostic criteria of SO differ substantially, and its impact on prediction of CVD, including mortality, significantly differs accordingly [54]. The recently introduced European Society for Clinical Nutrition and Metabolism and the European Association for the Study of Obesity (ESPEN-EASO-defined SO) tool, which uses weight-adjusted skeletal muscle mass assessment, showed the strongest associations with incident CVD and all-cause mortality (HR 1.61, 95% CI 1.43 to 1.81 and 1.66, 1.45 to 1.91, respectively) compared to older SO assessment tools [53]. The possible explanation of the superiority of the ESPEN-EASO-defined SO is its better capture of the muscle mass–adipose tissue mass metabolic imbalance. Sarcopenia and frailty significantly overlap, and indeed, sarcopenia is usually the biological substrate for frailty [55]. Therefore, assessment for CV risk should combine screening for sarcopenia and frailty, as they are both involved in CV risk prediction. Data from China Health and Retirement Longitudinal Study (CHARLS) found that the coexistence of sarcopenia and frailty was associated with the highest risk of incident CVD (HR 2.18, 95% CI 1.81 to 2.62) compared with sarcopenia or frailty alone. In addition, frailty mediated 35.4% of the association between sarcopenia and CVD [3].

Sarcopenia and frailty are not categorical conditions, but they are a continuum of a spectrum depending on body weight, which affects their metabolic profile and therefore their CV risk potential. The frailty metabolic profile was not explored in the studies, and frail participants were grouped as a single category. This led to variations in the results of the relationship between frailty and CVD. Studies with positive association included frail participants who were overweight or obese, whereas those showing no CVD risk included frail subjects with low body weight and less prevalence of CV risk factors. In addition to body composition, frailty appeared not to increase CV risk in the very old (≥90 years), compared with less old subjects (65–89 years) [56]. This may be due to physiologic redundancy and adaptive mechanisms that occur in very old age, which slow down the increase in the risk of mortality [57]. This has also been demonstrated in another study, which included frail participants aged ≥80 years and concluded that frailty could not be used in the very elderly to further identify additional individuals who might benefit more from CV risk management. Importantly, frail subjects in that study had no significant differences in their BMI, metabolic profile or CV risk factors compared with non-frail participants [58]. The SO or frail obese classes emerge as the phenotype with highest CV risk, while the isolated muscle mass decline or unintentional weight loss is the phenotype with least CV risk. Therefore, identification of these phenotypes is critical to precisely target and intensify intervention to reduce CV risk. For example, evidence from studies on sodium glucose cotransporter-2 (SGLT-2) inhibitors and glucagon-like peptide-1 receptor agonists (GLP-1RA) showed that CV risk protection was seen in the frail overweight or obese patients, and the SO phenotype stood to gain the most benefit. The SO phenotype had an unfavourable metabolic profile and higher baseline CV risk, and therefore benefited most from these therapies with the highest risk reduction and the lowest number needed to treat (NNT) compared with non-frail subjects. These therapies were not suitable, however, in those AM frail subjects due to low BMI, dehydration, hypotension and acute kidney injury [59]. Similarly, intensification of CV risk reduction medications should be aimed for in the SO due to progression of metabolic syndrome, while deintensification of therapy is appropriate in the AM phenotype due to regression of metabolic syndrome [60]. In the AM frail patients, all-cause rather than cardiac mortality is more prevalent, as these patients are likely to have multiple morbidities and more competing causes for death rather than just CVD. The use of invasive cardiac therapies may not necessarily be sufficient to improve prognosis, and such patients may benefit from more conservative and comprehensive geriatric care [38]. As obesity augments the negative effects of frailty or sarcopenia, every effort should be made to adopt a healthy lifestyle to avoid the development of SO. SO not only increases CV risk but is also associated with an increased prevalence of CV risk factors such as diabetes mellitus and CKD [61,62]. Physical activity and avoiding a sedentary lifestyle are essential to preserve muscle mass and reduce obesity. CV risk was shown to be reduced from 23% to 18% after adjusting for physical activity levels. This suggests that SO may promote sedentary behaviour and worsen cardiometabolic profiles, thereby amplifying the CV risk [62]. Lifestyle changes, which lead to weight and fat loss, should be accompanied by resistance exercise training to avoid muscle mass loss and maintain muscle strength, as muscle strength is equally important as muscle mass to reduce the risk of CVD [5,63].

## 10. Conclusions

Current evidence suggests that unintentional weight loss, linked to AM frailty, or isolated weak handgrip strength without obesity, a feature of sarcopenia, had a low prevalence of CV risk factors and is less likely to increase the risk of CVD, whereas obese sarcopenia or obese frailty had a higher prevalence of metabolic syndrome and CV risk factors such as hypertension, diabetes, dyslipidaemia, smoking and sedentary lifestyle, which is more likely to increase the risk of CVD (Box 1). Every effort should be made to maintain physical activity, reduce body fat, and maintain muscle mass and strength to reduce the development of sarcopenia, frailty and obesity and hence CVD. Aggressive treatment of SO is required with intensification of therapy to reduce the high CV risk encountered in these patients. Anorexic sarcopenic or frail subjects are likely to have multiple morbidities that compete for increasing cardiac mortality. A conservative and comprehensive geriatric approach with deintensification of therapy is appropriate in this vulnerable group of patients.

Box 1 **Status of this work.**

What is already known:

- Current studies suggest that sarcopenia and frailty are associated with an increased risk of cardiovascular disease.
- Current evidence considers sarcopenia or frailty as a single homogenous category with equal cardiovascular risk among sarcopenic frail individuals.

What this review adds:

- This review shows that the evidence of cardiovascular risk associated with sarcopenia or frailty occurs mostly in participants who are either over-weight or obese.
- This review suggests that sarcopenia–frailty is a heterogenous syndrome that spans across a metabolic spectrum with variation in the cardiovascular risk:
  a. The sarcopenic obese frail end of the spectrum has the highest cardi-ovascular risk due to elevated insulin resistance and high prevalence of cardiovascular risk factors.
  b. The anorexic malnourished frail end of the spectrum has the lowest cardiovascular risk due to reduced insulin resistance and low preva-lence of cardiovascular risk factors.

## 11. Future Perspectives

Recently published studies suggest that sarcopenia- or frailty-related CV risk is highest when obesity is present, which has also been previously reported [52,64,65,66]. However, some of the studies are limited by a cross-sectional or retrospective design, which investigates association rather than causation. In addition, some prospective studies have small sample sizes or a short duration of follow-up. Furthermore, the definition and diagnosis of sarcopenia varied across studies, the metabolic phenotypes of frailty were not explored and frailty was considered as a single homogenous category despite the heterogeneous nature of this condition. The presence of a metabolic spectrum of frailty may explain the phenomenon of the obesity paradox. It is plausible that the apparent protective effect of obesity is actually due to the increased mortality at the AM end of the frailty spectrum, because significant anorexia, malnutrition and weight loss lead to increased risk of all-cause mortality, but this needs future exploration [67]. The emergence of sarcopenia–frailty as a novel combined CV risk factor will need substantial research for the clarification of reliable diagnostic methods. Future exploration of the characteristics of which metabolic phenotype is at highest risk of CVD and the preventative strategies is required. For example, there is a gap between the field of sarcopenia and routine clinical practice, as the majority of clinicians are unaware of the condition, its diagnostic methodology and the link between sarcopenia and metabolic dysregulation that increases the CV risk [68]. Future directions in sarcopenia research, such as the adiposity-adjusted muscle mass assessment, will improve its prognostic potential. By normalising the ratio of muscle mass to total body weight, the ESPENEASO framework may better capture the disproportion between fat and muscle that underlies cardiometabolic vulnerability [54]. The recently introduced triglyceride-glucose (TyG) and TyG-body mass index (TyG-BMI) are increasingly used as surrogate markers of insulin resistance and have been shown to improve the stratification of mortality risk in different ethnic groups. This supports their potential utility as accessible and cost-effective tools for metabolic risk assessment in diverse populations [69]. In addition, not many studies investigated the effects of central, as opposed to general, obesity, which seems to have a greater impact on metabolic abnormalities and CV risk [70]. Lack of anthropometric measurements of central obesity such as waist-to-hip ratio, nutritional intake or physical activity may act as confounders, which might have influenced the results of the current studies and need to be explored in future research.

There are still substantial deficiencies in our knowledge about frailty syndrome, its metabolic heterogeneity and therefore, its variable impact on CV risk. Establishing a robust definition and exploring metabolic characteristics of frail subjects is essential from the outset, before inclusion in future studies. This should improve the study design and allow a better comparison between clinical trials and therefore application in clinical practice. Similar to sarcopenia studies, other unmeasured confounding factors may have influenced the association of frailty with CVD, such as hypovitaminosis D, depression or chronic obstructive pulmonary disease, as these conditions, especially depression, may play a role in the pathogenesis of CVD [71,72,73]. In addition, adherence to medications was not always checked in frailty studies, as frailty is associated with poor medication compliance, which may increase their risk of CVD [74]. Sarcopenia increases the risk of frailty and its progression to disability [75]. These two conditions often coexist and influence each other; however, current studies have mostly focused on the individual effects of either condition, overlooking the combined impact they may have on CVD risk [76]. As sarcopenia and frailty significantly overlap, an approach that combines both conditions together appears appropriate. This should focus on exploration of the main molecular pathways and pathologies that mediate both conditions, thereby enabling the identification of appropriate molecular targets for the development of specific therapies. From an epidemiologic, pathologic, prognostic and pragmatic point of view, it appears more appropriate to address both conditions as one, sarcopenia–frailty syndrome. Evidence suggests that this sarcopenia–frailty syndrome can be reversed with timely intervention [77,78]. Therefore, future and practical ways of monitoring the transitions in sarcopenia–frailty syndrome over time may enable early identification and intervention in individuals at risk of CVD before irreversible damage occurs. Addressing these priorities may provide the foundation for future prevention of this increasingly debilitating syndrome.

## 12. Key Points from This Narrative Review

- Sarcopenia and frailty are not discrete, isolated diagnoses as they represent a progressive structural–functional–metabolic pathway.
- Both sarcopenia and frailty are associated with an increased risk of cardiovascular disease.
- Sarcopenia and frailty significantly overlap and may represent a biological continuum. Together, they augment cardiovascular risk more than either condition alone.
- Obesity-associated sarcopenia or frailty imposes the highest risk, while sarcopenia alone or the anorexic-malnourished frailty metabolic phenotype appears to be less linked with cardiovascular risk.
- Unfavourable metabolic profiles and prevalent cardiovascular risk factors are likely the mediators of cardiovascular risk in obesity-associated sarcopenia or frailty.
- Future research in cardiovascular disease should consider the combined sarcopenia–frailty syndrome as a key area to further investigate common pathogenesis and ways of prevention.

## Figures and Tables

**Table 1:** Studies reported high CV risk in overweight or obese sarcopenic or frail patients.

Study	Population	Aim to	Body Composition	Outcomes
**Yu Z**, et al., prospective, China, 2025. [22]	7702 subjects, mean age 39 Y, 690 sarcopenic, 7012 non-sarcopenic.	Explore relation between sarcopenia and CVD.	Sarcopenic vs. non-sarcopenic: Median (IQR) age 44 (33, 54) vs. 38 (28, 48) Y, *p* < 0.001, Mean (SD) BMI 34.25 (7.97) vs. 28.24 (6.43), *p* < 0.001, DM 15.1% vs. 6.7%, *p* < 0.001, HTN 61.3% vs. 73.4%, *p* < 0.001, CKD 3.3% vs. 1.7%, *p* < 0.005, dyslipidaemia 32% vs. 23.2%, *p* < 0.001, CVD 6.9% vs. 3.3%, *p* < 0.001.	Sarcopenia associated with CVD, OR 1.89 (95% CI 1.04 to 3.43, *p* = 0.03), HRs for cardiovascular and all-cause mortality 1.95 (95% CI, 0.62 to 6.12, *p* = 0.25 and 1.43 (0.71 to 2.87, *p* = 0.32), respectively.
**Boonpor J**, et al., prospective, UK, 2024. [23]	11,974 subjects, mean (SD) age 59.8 (7) Y, F/U 10.7Y.	Investigate associations of sarcopenia with CVD.	Sarcopenia vs. non-sarcopenia: mean (SD) BMI: 33.9 (6.6) vs. 30.6 (5.1), sedentary time 6.2 (3) vs. 5.7 (2.4) hour/day, DM duration 10.3 (11.3) vs. 8.4 (10.1) Y.	Sarcopenia increased risk of CVD, HR 1.89 (95% CI 1.61 to 2.21), HF 2.59 (2.12 to 3.18), stroke 1.90 (1.38 to 2.63), MI 1.56 (1.04 to 2.33) adjusted for covariates.
**Xin Y**, et al., prospective, China, 2025. [24]	6766 subjects, mean (SD) age 60.0 (9.9) Y.	Explore association of SO and advanced CKM syndrome.	Possible sarcopenia or sarcopenia in NW vs. OW categories: HTN 55.8% vs. 73.6%, *p* < 0.001, DM 15.8% vs. 24.6%, *p* < 0.001, MetS 27.5% vs. 73.6%, *p* < 0.001, BMI 20.7 (2.1) vs. 27.1 (3.0), *p* < 0.001, WC 78.7 (10.4) vs. 92.9 (12.5), *p* < 0.001.	After F/U 9.0 Y: SO associated with increased risk of MACEs, HR 2.25 (95% CI 1.79 to 2.82). Sarcopenic overweight 1.77 (1.47 to 2.14) and sarcopenic abdominal obesity 1.73 (1.41 to 2.12) associated with elevated risk of MACEs.
**Jiang M**, et al., prospective, China, 2024. [25]	7703 ≥ 45 Y. F/U 7 Y.	Examine effects of SO and possible SO on CVD.	Obesity defined BMI ≥ 28 or WC ≥ 85 cm males and ≥80 cm females.	SO increased risk of CVD, HR 1.39 (95% CI 1.16 to 1.67), heart disease 1.36 (1.10 to1.67) and stroke 1.40 (1.02 to 1.92).
**Chuan F**, et al., retrospective, China, 2022. [26]	386 subjects with type 2 DM, mean (SD) age 67.9 (6.1) Y.	Investigate impact of SO on negative health outcomes.	SO defined as coexistence of sarcopenia defined by 2019 Asian Working Group for Sarcopenia up-to-date consensus and obesity identified by five alternative measurements: BMI ≥ 28 (BMI28), BMI ≥ 25 (BMI25), BF% ≥ 25% for men or 35% for women, VFA ≥ 100 cm^2^ or AF mass higher than the sex-specific median.	A. SO classified using BF% significantly associated with incident CVD, HR 6.02 (95% CI 1.56 to 23.15) compared with either sarcopenia or obesity alone. B. SO classified using BMI25 resulted in misclassification of SO.
**Fukuda T**, et al., retrospective, Japan, 2018. [27]	716 subjects, with type 2 DM, mean (SD) age 65.0 (13) Y, F/U 2.6 Y.	Investigate impact of SO on incident CVD.	Obese, sarcopenia, SO: mean (SD) BMI: 29.1 (5.3), 20.8 (2.9), 23.7 (3.0), *p* < 0.001, BF mass: 28.1% (7.9), 38.8% (6.4), 35.3% (5.3), *p* < 0.001, DM duration: 10.7 (8.8), 11.4 (10.9), 15.3 (13.1) Y.	SO associated with incident CVD when using A/G ratio, HR 2.63 (95% CI 1.10 to 6.28, *p* = 0.030) and android fat mass 2.57 (1.01 to 6.54, *p* = 0.048) to define obesity, but not BF or BMI.
**Farmer RE**, et al., cohort biobank, UK, 2019. [28]	Using UK Biobank of 452,931 patients, mean age range 56.2 to 59.5 Y.	Investigate associations of SO and CVD risk.	Obese, sarcopenia, SO: mean (SD) BMI: 33.81 (3.74), 25.19 (2.82), 34.37 (4.74), BF mass: 37.62% (7.75), 31.83% (7.31), 41.81% (6.98), WHR: 0.92 (0.09), 0.85 (0.08), 0.91 (0.09), DM: 9.8%, 4.6%, 15.4%.	Outcomes: fatal and non-fatal CVD and mortality. A. Obesity associated with increased risk of all outcomes, HR range 1.10–1.82. B. Adverse effect of obesity on outcomes was not reduced by improved muscle quality.
**Hannan M**, et al., prospective, US, 2024. [29]	2539 participants, mean (SD) age 62.0 (10.5) Y. median F/U 11.4 Y.	Assess relation of frailty with CV outcomes.	Frail-Pre-frail, Robust: Mean (SD) BMI: 35 (9), 33 (8), 30 (6), eGFR: 36.5 (0.98), 42.2 (0.51), 52.5 (0.66), DM: 65%, 54%, 36%, HTN: 98%, 94%, 86%, CVD: 52%, 44%, 24%, statin use: 64%, 67%, 59%.	Frailty, pre-frailty increased risk of CV events, HR 2.03 (95% CI 1.41 to 2.91) and 1.77 (1.35 to 2.31), HF 2.22 (1.59 to 3.10) and 1.39 (1.07 to 1.82), all-cause and CV mortality 2.52 (1.84 to 3.45), 1.76 (1.37 to 2.24), 3.01 (1.62 to 5.62) and 1.78 (1.06 to 2.99), respectively.
**He D**, et al., prospective, China, 2024. [30]	10,172 subjects from CHARLS (mean age: 57.8 Y), 6448 from ELSA (64.1 Y), 9427 from HRS (66.1 Y).	Explore frailty status changes and incident CVD.	Baseline: Frail vs. robust: CHARLS: BMI 24.3 (3.9) vs. 23.1 (3.7). ELSA: BMI 29.8 (5.8) vs. 27.3 (4.4). HRS: BMI 30.1 (6.9) vs. 26.5 (4.4).	A. Progression of robust to pre-frail or frail ↑ risks of CVD (CHARLS, HR 1.84 (95% CI 1.54 to 2.21), ELSA 1.53 (1.25 to 1.86), HRS 1.59 (1.31 to 1.92). B. Recovery of frail to robust or pre-frail ↓ risks of CVD, CHARLS 0.62 (0.47 to 0.81), ELSA 0.49 (0.34 to 0.69), HRS 0.70 (0.55 to 0.89). C. Recovery of pre-frail to robust ↓ risks of CVD, CHARLS 0.66, (0.52 to 0.83), ELSA 0.65 (0.49 to 0.85), HRS 0.71 (0.56 to 0.91).
**Chen L**, et al., prospective, China, 2023. [31]	314,093 UK biobank participants, mean (SD) age 55.9 (8.1), F/U 11.3 (2.2) Y.	Investigate relation of frailty with risk of CVD.	Respectively, frail-pre-frail, robust: Mean (SD) BMI: 30.8 (6.5), 27.9 (4.9), 26.3 (4.0), medication use for HTN 32.4%, 19.5%, 13.9%, for lipid lowering 26.0%, 15.1%, 10.7%, for DM 8.3%, 2.9%, 1.1%.	A. Robust incident rate of CVD 6.54 per 1000 person-years. B. Absolute rate difference per 1000 person-years 1.67 (95% CI 1.33 to 2.02) for pre-frail and 5.0 (4.03 to 5.97) for frail.
**Damluji AA**, et al., prospective, US, 2021. [32]	3259 participants, mean age 77.6 Y, F/U 6 Y.	Explore relation of frailty with MACE and all-cause mortality.	Respectively frail, pre-frail, robust: BMI: 26.5, 27.5, 26.9, *p* = 0.001. DM: 30.8%, 21.5%, 16.5%, *p* < 0.001. HTN: 71.3%, 66.8%, 56.6%, *p* < 0.001.	MACE higher in frail than robust, HR 1.77 (95% CI 1.53 to 2.06), death 2.70 (2.16 to 3.38), MI 1.95 (1.31 to 2.90), stroke 1.71 (1.34 to 2.17), PVD 1.80 (1.44 to 2.27), CHD 1.35 (1.11 to 1.65).
**Veronese N**, et al., prospective, Italy, 2017. [33]	3818 subjects, mean (SD) age 76.2 (5.6) Y, median F/U 8.7 Y.	Evaluate effect of frailty on CVD risk.	Frail compared to non-frail: Mean (SD) BMI 28.1 (5.3) vs. 26.9 (4.4), *p* < 0.0001, WC 104.3 (13.3) vs. 100 (12) cm, *p* < 0.0001, DM 14.8% vs. 10.9%, *p* < 0.01, MetS 36.9% vs. 28.4%, *p* < 0.0001, use of antihypertensives 57.6 vs. 46.2%, *p* < 0.0001.	Frailty increased risk of CVD, HR 1.35 (95% CI 1.05 to 1.74) after adjusting for clinical, biochemical and subclinical atherosclerotic disease.
**Fan J**, et al., prospective, China, 2020. [34]	512,723 subjects, mean (SD) age 52.0 (10.7) Y, median F/U 10.8 Y.	Explore frailty effect on mortality.	Respectively frail-pre-frail, robust: BMI < 18.5 or >28: 38.6%, 24.8%, 7.2%, WHR: ≥0.95 men/≥0.90 women: 58.0%, 45.8%, 17.4%, HTN: 67.2%, 52.8%, 20.2%, DM: 26.7%, 10.0%, 1.3%.	Each 0.1 increment in FI increased risk of all-cause mortality, HR 1.68 (95% CI 1.66 to1.71), death from CVD 1.89 (1.83 to 1.94) and cerebrovascular disease 1.84 (1.79 to 1.89).

Y = years, CVD = cardiovascular disease, IQR = interquartile range, SD = standard deviation, BMI = body mass index, DM = diabetes mellitus, HTN = hypertension, CKD = chronic kidney disease, OR = odds ratio, CI = confidence interval, HR = hazard ratio, F/U = follow up, HF = heart failure, MI = myocardial infarction, SO = sarcopenic obesity, CKM = cardiovascular kidney metabolic, NW = normal weight, OW = overweight, MetS = metabolic syndrome, WC = waist circumference, MACE = major adverse cardiovascular events, BF = body fat, VFA = visceral fat area, AF = android fat, A/G = android to gynoid, WHR = waist hip ratio, eGFR = estimated glomerular filtration rate, CHARLS = Chinese health and retirement longitudinal study, ELSA = English longitudinal study of ageing, HRS = health and retirement study, PVD = peripheral vascular disease, CHD = coronary heart disease. ↑ = increased, ↓ = decreased.

**Table 2:** Studies reported low CV risk in underweight sarcopenic or frail patients.

Study	Population	Aim to	Body Composition	Outcomes
**Chen Y**, et al., prospective, China, 2025. [35]	10,649 subjects, mean (SD) age 64.5 (10.7) Y, F/U 1.6 (1.1) Y.	Analyse whether sarcopenia is associated with new onset CVD.	No sarcopenia, possible sarcopenia, sarcopenia, severe sarcopenia: Age ≥ 70 Y: 19%, 46.6%, 67.2%, 86.6%, *p* < 0.001, median (IQR) BMI: 24.1 (22.3, 26.3), 24.6 (22.9, 26.7), 19.7 (18.6, 20.5), 19.4 (17.5, 20.6), *p* < 0.001, underweight: 2.2%, 0%, 25%, 33%, *p* < 0.001, obesity: 17%, 20.5%, 0%, 0%, *p* < 0.001, abdominal obesity: 58%, 69%, 18.3%, 21.5%, *p* < 0.001, HTN: 14.8%, 25%, 13.6%, 18.2%, *p* < 0.001, dyslipidaemia: 6.9%, 8.1%, 3.3%, 3.4%, *p* < 0.001, DM: 3.7%, 6.2%, 3.4%, 2.6%, *p* < 0.001.	A. Possible sarcopenia increased new onset CVD, HR 1.21 (95% CI, 1.06 to 1.37). B. Sarcopenia and severe sarcopenia showed no association. C. Longer 5-CST linked to higher risk of new onset CVD.
**Zeng Q**, et al., prospective, China, 2024. [36]	7499 subjects, mean (SD) age 58.5 (9.2) Y. F/U 7Y.	Investigate changes in sarcopenia status and incidence of CVD.	No sarcopenia vs. possible sarcopenia vs. sarcopenia: A. Baseline data: BMI: 23.7 (3.7), 24.7 (3.5), 19.1 (1.8), *p* < 0.0001, DM: 11.5%, 13.9%, 8.7%, *p* < 0.001, HTN: 34%, 45.5%, 41.2%, *p* < 0.0001. B. Prospective data: BMI: 23.7 (3.7), 24.7 (3.5), 19.1 (1.8), *p* < 0.0001, DM: 11.7%, 14.1%, 6.9%, *p* < 0.001, HTN: 33.5%, 44.8%, 36%, *p* < 0.0001.	A. Baseline: possible sarcopenia increased risk of CVD compared to no sarcopenia, HR 1.25 (95% CI 1.11 to 1.42). Sarcopenia did not significantly increase risk of CVD 1.01 (0.81 to 1.26). B. Prospective: possible sarcopenia increased risk of CVD compared to no sarcopenia, HR 1.30 (95% CI 1.06 to 1.59). Possible sarcopenia progressed to sarcopenia did not show significant risk of CVD.
**Gao K**, et al., cross sectional/prospective, China, 2022. [37]	15,137 subjects, mean (SD) age 60.6 (9.9) Y, 3.6 Y, F/U.	Investigate association between sarcopenia status and CVD.	Sarcopenic vs. non-sarcopenic: mean (SD) age: 68.5 (10.4) vs. 58.0 (8.5), *p* < 0.001, BMI: 20.9 (3.7) vs. 24.1 (3.6), *p* < 0.001, HTN: 34.5% vs. 29.3%, *p* < 0.001, Dyslipidaemia: 9.6% vs. 12.1%, *p* < 0.001, DM: 9.3% vs. 7.7%, *p* < 0.001, CKD 7.9% vs. 5.7%, *p* < 0.001.	A. Sarcopenia significantly associated with CVD in cross sectional and prospective analyses OR 1.72 (95% CI 1.40 to 2.10) and 1.33 (1.04 to 1.71), respectively. B. Low muscle mass alone or underweight not associated with CVD in cross sectional or prospective analysis.
**Patel A**, et al., prospective, Australia, 2018. [38]	3944 subjects ≥ 65 Y, 1275 STEMI, 2669 non-STEMI.	Investigate effects of frailty after AMI.	Frail vs. non-frail: A. STEMI: Median (IQR) weight 75 (62, 87) vs. 78 (68, 87), *p* = 0.37, DM 48.4% vs. 19.1%, *p* < 0.001, HTN 88% vs. 57.7%, *p* < 0.001, dyslipidaemia 81.8% vs. 41.8%, *p* < 0.001. B. Non-STEMI: weight 79 (68, 92) vs. 78 (68, 90) *p* = 0.49, DM 53.2% vs. 23.9%, *p* < 0.001, HTN 90.8% vs. 66.2%, *p* < 0.001, dyslipidaemia 84.1% vs. 52.3%, *p* < 0.001.	A. FI increased all-cause in-hospital mortality, OR 1.38 per 0.1 FI (95% CI 1.05 to 1.83, *p* = 0.02) but not cardiac mortality. B. FI increased 6-month all-cause but not cardiac mortality, STEMI, OR 1.74 (1.37 to 2.22, *p* < 0.001), non-STEMI, 1.62 (1.40 to 1.87, *p* < 0.001).
**Kleipool EE**, et al., prospective, Netherland, 2018. [39]	1284 subjects aged 65–88 Y, F/U 8.4 Y.	Investigate bidirectional association of frailty and CVD.	Frail vs. non-frail: BMI < 20: 8% vs. 4%, BMI 20–25: 27% vs. 31%, BMI > 25: 65% vs. 65%, *p* = 0.04, mean (SD), total cholesterol: 5.5 (1.2) vs. 5.7 (1.0), *p* = 0.02, LDL: 3.5 (1.1) vs. 3.7 (0.9), *p* = 0.02.	Frailty was not significantly associated with incident CVD, HR 1.47 (95% CI 0.86 to 2.52).
**Zhu X**, et al., prospective, China, 2024. [40]	8512 subjects, mean (SD) age 58.63 (9.18) Y.	Investigate relation of PF, circS and CVD.	Mean (SD) in PF, circS, both and non-frail respectively: BMI: 19.72 (3.27), 25.56 (3.68), 23.34 (4.56), 22.7 (3.36), *p* < 0.001. UW: 46.5%, 0.6%, 16.2%, 3.7%, *p* < 0.001. NW: 44.3%, 34.4%, 41.4%, 67.8%, *p* < 0.001. OW: 9.2%, 64.9%, 42.4%, 28.5%, *p* < 0.001. HTN: 15%, 41.1%%, 47.4%, 10.8%, *p* < 0.001. DM: 4.3%, 11.1%, 15.7%, 2.2%, *p* < 0.001.	A. CircS more likely to be frail OR 2.07 (95% CI 1.732 to 2.47). C. CircS 1.954 (1.66 to 2.30), and CircS plus PF 3.508 (2.74 to 4.49) associated with CVD. D. CircS plus PF 1.72 (1.31 to 2.24) and CircS 1.520 (1.33 to 1.74) more likely to develop new onset CVD.

SD = standard deviation, Y = years, F/U = follow up, CVD = cardiovascular disease, IQR = interquartile range, BMI = body mass index, HTN = hypertension, DM = diabetes mellitus, HR = hazard ratio, CI = confidence interval, 5-CST = 5 times chair stand test, CKD = chronic kidney disease, OR = odds ratio, STEMI = ST-elevation myocardial infarction, AMI = acute myocardial infarction, FI = frailty index, LDL = low density lipoprotein, PF = physical frailty, circS = circadian syndrome, UW = underweight, NW = normal weight, OW = overweight.

## Data Availability

No new data were created or analyzed in this study. Data sharing is not applicable to this article.
